# Profiling of *in vivo*, *in vitro* and reactive zorifertinib metabolites using liquid chromatography ion trap mass spectrometry†

**DOI:** 10.1039/d2ra02848d

**Published:** 2022-07-21

**Authors:** Nasser S. Al-Shakliah, Adnan A. Kadi, Haya I. Aljohar, Haitham AlRabiah, Mohamed W. Attwa

**Affiliations:** Department of Pharmaceutical Chemistry, College of Pharmacy, King Saud University P.O. Box 2457 Riyadh 11451 Saudi Arabia mzeidan@ksu.edu.sa +966 1146 76 220 +966 1146 70237

## Abstract

Zorifertinib (AZD-3759; ZFB) is a potent, novel, oral, small molecule used for the treatment of non-small cell lung cancer (NSCLC). ZFB is Epidermal Growth Factor Receptor (EGFR) inhibitor that is characterized by good permeability of the blood–brain barrier for (NSCLC) patients with EGFR mutations. The present research reports the profiling of *in vitro*, *in vivo* and reactive metabolites of ZFB. Prediction of vulnerable metabolic sites and reactivity pathways (cyanide and GSH) of ZFB were performed by WhichP450™ module (StarDrop software package) and XenoSite reactivity model (XenoSite Web Predictor-Home), respectively. ZFB *in vitro* metabolites were done by incubation with isolated perfused rat liver hepatocytes and rat liver microsomes (RLMs). Extraction of ZFB and its related metabolites from the incubation matrix was done by protein precipitation. *In vivo* metabolism was performed by giving ZFB (10 mg kg^−1^) through oral gavage to Sprague Dawley rats that were housed in metabolic cages. Urine was collected at specific time intervals (0, 6, 12, 18, 24, 48, 72, 96 and 120 h) from ZFB dosing. The collected urine samples were filtered then stored at −70 °C. *N*-Methyl piperazine ring of ZFB undergoes phase I metabolism forming iminium intermediates that were stabilized using potassium cyanide as a trapping agent. Incubation of ZFB with RLMs were performed in the presence of 1.0 mM KCN and 1.0 mM glutathione to check reactive intermediates as it is may be responsible for toxicities associated with ZFB usage. For *in vitro* metabolites there were six *in vitro* phase I metabolites, three *in vitro* phase II metabolites, seven reactive intermediates (four GSH conjugates and three cyano adducts) of ZFB were detected by LC-IT-MS. For *in vivo* metabolites there were six *in vivo* phase I and three *in vivo* phase II metabolites of ZFB were detected by LC-IT-MS. *In vitro* and *in vivo* phase I metabolic pathways were *N*-demethylation, *O*-demethylation, hydroxylation, reduction, defluorination and dechlorination. *In vivo* phase II metabolic reaction was direct sulphate and glucuronic acid conjugation with ZFB.

## Abstract 2

Six *in vitro* phase I metabolites, three *in vitro* phase II metabolites, seven reactive intermediates (four GSH conjugates and three cyano adducts), six *in vivo* phase I and three *in vivo* phase II metabolites of ZFB were detected by LC-IT-MS.

## Graphical abstract

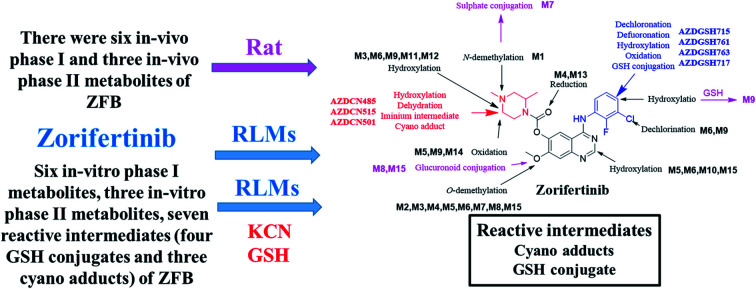

## 1. Introduction

Non-small-cell lung carcinoma (NSCLC) is any type of epithelial lung cancer other than small cell lung carcinoma (SCLC). NSCLC accounts for about 85% of all lung cancers.^1^ NSCLC treatment had a breakthrough in the past 15 years with tyrosine kinase inhibitors, specifically with the first generation gefitinib and erlotinib and, currently, with the second generation afatinib. These agents are referred to as “targeted” therapy since they target epidermal growth factor (EGF) mutations in lung cancer adenocarcinoma patients.^2–4^ EGF is a single polypeptide of fifty-three amino acid residues which is contributed in the cell proliferation regulation. EGF and EGFR play a crucial role in wound healing through stimulating dermal and epidermal regeneration.

The new therapies development for improving wound healing has involved the usage of EGF. In addition, EGFR inhibitors have become a way for the cancer treatment. Thus, therapies targeting EGF/EGFR are suitable for the treatment of cancer and cutaneous wounds.^5^ The first agent developed as a target cancer therapy was EGFR inhibitor. There are two classes of EGFR inhibitors are in current use: the monoclonal antibodies (cetuximab, panitumumab, and matuzumab) that target the extracellular ligand-binding domain and small-molecule tyrosine kinase inhibitors such as ZFB which target intracellular domain.^6,7^

ZFB is a novel small molecule used for NSCLC treatment through inhibition of EGFR and characterized by good permeability of the blood–brain barrier (BBB) for (NSCLC) patients with EGFR mutations.^8^ ZFB can penetrate the BBB and was confirmed to be effective in both clinical and preclinical settings, and showed promising activity in patients with central nervous system (CNS) metastasis as monotherapy.^8,9^ ZFB is currently at a phase II/III clinical trials.^10^ Side effects of ZFB was skin and gastrointestinal disorders of occurred in 92% and 76% patients respectively. Other disorders included hepatobiliary and renal disorders (13%), asthenia (7%), infections and infestations (7%), and metabolism and nutrition disorders at 4% and 7%, respectively.^11^

ZFB chemical name is 4-((3-chloro-2-fluorophenyl)amino)-7-methoxyquinazolin-6-yl (*R*)-2,4-dimethylpiperazine-1-carboxylate. ZFB chemical structure contains *N*-methyl piperazine and phenyl amine rings that could undergo metabolic bioactivation generating iminium and iminoquinone reactive intermediate, respectively that can be captured by nucleophile such as potassium cyanide (KCN) and GSH, respectively forming cyano adducts and GSH conjugates, respectively that are stable and could be identified by liquid chromatography ion trap mass spectrometry (LC-IT-MS). Extraction, identification, separation and characterization of these stabilized adducts and conjugates were performed using LC-IT-MS.^12–14^ These reactive metabolites helped us to predict toxicities associated with ZFB.

Our research team previously established LC-MS/MS analytical method for ZFB quantification with application to assess its metabolic stability which revealed the moderate extraction ratio and the good bioavailability of ZFB.^15,16^ This research will focus on the profiling of ZFB metabolites either *in vitro* or *in vivo* using LC-IT-MS. Metabolic bioactivation of a drug to reactive intermediates that can covalently modify proteins leading to drug-induced organ toxicities. Hence, we will check the reactive intermediates that will aid in predicting the reasons for toxicities associated with these drugs. Reactive intermediates cannot be checked *in vivo* as a result of as once they are formed, they will bind to endogenous materials as proteins or DNA that prevents the detection by LC-IT-MS.^17^

## 2. Chemicals and methods

### 2.1. Chemicals and instruments

Zorifertinib was purchased from Med Chem Express LLC company (Princeton, NJ, USA). Reference powders (analytical grade) and solvents (HPLC grade) were used. Sprague Dawley rats were obtained from Health Research Center of Prince Naif bin Abdul Aziz at King Saud University (Riyadh, KSA). Rat liver microsomes (RLMs) were prepared in our laboratory following a published protocol using Sprague Dawley rats.^18,19^ Acetonitrile (ACN), nicotinamide adenine dinucleotide phosphate (NADPH), formic acid (HCOOH), potassium cyanide (KCN) and glutathione reductase (GSH) were purchased from Sigma-Aldrich company (St. Louis, MO, USA). Water (HPLC grade) was supplied by Milli-Q plus purification system (Billerica, MA, USA) that is available in-house. Tween 80 were obtained from Eurostar Scientific Ltd. (Liverpool, UK). Ethical approval for the Animal experiments of were obtained from the Animal Ethics Committee at King Saud University (No. KSU-SE-19-52). The separation of ZFB and its related metabolites and adducts was done on an Agilent HPLC 1200 series that consists of G1367B HIP-ALS auto sampler, G1322A degasser, G1316 thermostated column compartment and G1311A binary pump that was connected using an electrospray ionization (ESI) to an Agilent 6320 Ion Trap (Agilent Technologies, Palo Alto, CA, USA). Mass Hunter software (Agilent Technologies, Palo Alto, CA, USA) was used to regulate the data acquisition and instruments.

### 2.2. Chromatographic conditions

The analytical parameters that were used for separation of ZFB and its related metabolites are listed are mentioned in Table 1. Agilent eclipse plus C18 analytical column (150 mm × 2.1 mm, 3.5 μm particle size) (Agilent Technologies, Palo Alto, CA, USA). Column temperature was kept constant at 23 ± 2 °C. The most appropriate chromatographic conditions were attained at a flow rate of 0.25 mL min^−1^ with a gradient system for 65 min. Mobile phase composed of 0.1% HCOOH (pH: 3.2) and ACN. The post time was 15 min. Sample injection volume was 10 μL. Mass parameters were optimized for ZFB. Dissociation for ZFB and related metabolites was done using collision-induced dissociation (CID) in the collision cell.

**Table 1:** Adjusted parameters of the proposed LC-MS experiment

Liquid chromatographic parameters			Mass spectrometric parameters	
HPLC	Agilent 1200		Mass spectrometer	Agilent 6320 ion trap
Gradient mobile phase	A: 1% HCOOH in H_2_O		Ionization source	Positive ESI
	B: ACN			Drying gas: N_2_ gas
	Flow rate: 0.25 mL min^−1^			Flow rate (10 L min^−1^)
	Run time: 65 min			Pressure (60 psi)
Agilent eclipse plus C_18_ column	Length	150 mm		ESI temperature: 350 °C
	Internal diameter	2.1 mm		Capillary voltage: 4000 V
	Particle size	3.5 μm	Collision gas	High purity N_2_
	Temperature	23 ± 2 °C	Modes	Mass scan and MS^2^
Gradient system	Time	% B	Analytes	ZFB and its metabolites
	0	5		
	5	5	Mass parameters	
	30	30		Fragmentor voltage: 145 V
	40	40		Amplitude: 1.25 V
	50	80		
	60	90		
	65	5		

### 2.3. *In silico* ZFB metabolism prediction using WhichP450™ (StarDrop software)

Our target is to recognize the susceptibility of metabolism key sites, as shown by the low value of the site lability that was revealed by the composite site lability (CSL) and also the proposed major isoforms regioselectivity that was supposed to be responsible for metabolism. The results from the WhichP450™ software, displayed by the pie chart used for indication of most probably cyp450 isoform that has the major role in ZFB metabolism. These predictions were used as a director during profiling of ZFB *in vivo* and *in vitro* metabolites.^20,21^

### 2.4. *In silico* of ZFB reactive intermediates and toxicity prediction using XenoSite reactivity model and DEREK software

XenoSite reactivity model is freely available online at https://swami.wustl.edu/xenosite that was used to detect the labile sites that could produce reactive intermediates that could form complex with GSH, cyanide, DNA and protein stable adducts.^22–24^ This *in silico* model is based on neural networks of around 680 molecules.^25^ This free online software characterized by short running time depending on the molecule size.^21^ SMILES format (CC(C)Oc1ccc(cc1)NC(=O)N2CCN(CC2)c3c4cc(c(cc4ncn3)OCCCN5CCCCC5)OC) of ZFB was uploaded to XenoSite reactivity model that is an online website. The outcomes were used in searching and identifying reactive metabolites of ZFB.

DEREK module (StarDrop software package) was utilized for screening for ZFB and its related metabolites structural alerts and to verify our bioactivation pathway proposal. DEREK software can be used to propose structural modification at the bioactive sites that may stop the initiation of toxicity sequence. The chemical structure of ZFB and its related metabolites (SMILES format) was uploaded on the program software for the toxicity prediction.

### 2.5. *In vitro* metabolism

#### 2.5.1. RLMs incubations

RLMs were incubated with ZFB following a previously published procedure.^26,27^ Thirty μM ZFB in DMSO was added to 0.08 M phosphate buffer (pH 7.4) with RLMs (1 mg mL^−1^). The incubation mixture was first kept in a shaking water bath for 5 min at 37 °C. Incubation was initiated by NADPH (1 mM) solution. The final volume of incubation mixture is 1 mL and the total time of incubation is 90 min. Controls were prepared either by replacing RLMs or NAPDH with buffer. The metabolic reaction was stopped adding 2 mL ice-cold acetonitrile then centrifugation at 9000*g* for 10 min for protein precipitation. The supernatant was removed to clean vials for evaporation using a stream of nitrogen as inert gas. The residue was solubilized in a mixture of 0.1% HCOOH and ACN in a ratio similar to mobile phase and transferred to HPLC vial for analysis. To trap reactive intermediates, the same experiment was repeated using 1.0 mM KCN or 1.0 mM GSH. Similarly, negative controls were done using the above steps. Phenytoin (2 μM) was used to evaluate the activity of RLMs as the positive control.

#### 2.5.2. Liver hepatocytes incubation

Rat liver hepatocytes (RLHs) will be done following the established steps.^28^ RLHs incubation experiment with ZFB (30 μM) was performed in a rotary evaporation instrument fitted with a custom made 4-way adaptor to permit the connections of four 100 mL round bottom flasks. The RLHs incubations were performed at 37 °C for 2 hours. One mL aliquots (at 0, 15, 30, 60, 90, and 120 minutes were taken from each flask, mixed with 2 mL of ACN and immediately frozen at −70 °C refrigerator). Cell viability was retested using trypan blue at the end of the experiment. Stored frozen samples were thawed and then centrifuged at 14 000 rpm. Supernatants were transferred to new vials and evaporated under a stream of inert gas nitrogen. The residue was reconstituted in 300 μl of the mobile phase in HPLC vials for LC-MS analysis. Negative control experiments were done following the above steps.

### 2.6. *In vivo* metabolism

Four male one month Sprague-Dawley rats of average weight (400 g) were housed individually in a metabolism cage that was kept in animal care facility in a 12 h light/dark cycle (7:00–19:00). Rats had free access to standard animal food and water. Rats were kept in metabolism cages for 72 h before the start of the experiment. ZFB was solubilized in a special solution (4% DMSO, 5% Tween 80 and 30% PEG 300, in HPLC H_2_O) to allow ZFB dispersion and to maintain the drug solubility. The ZFB dose for rats was calculated by conversion from reported human dose to rat dose.^29^ Each rat received a specific dose of ZFB, depending on its weight. The ZFB dose for rat was 10 mg per kg per day.

## 3. Results and discussion

### 3.1. *In silico* prediction of ZFB metabolites

**Fig. 1:**
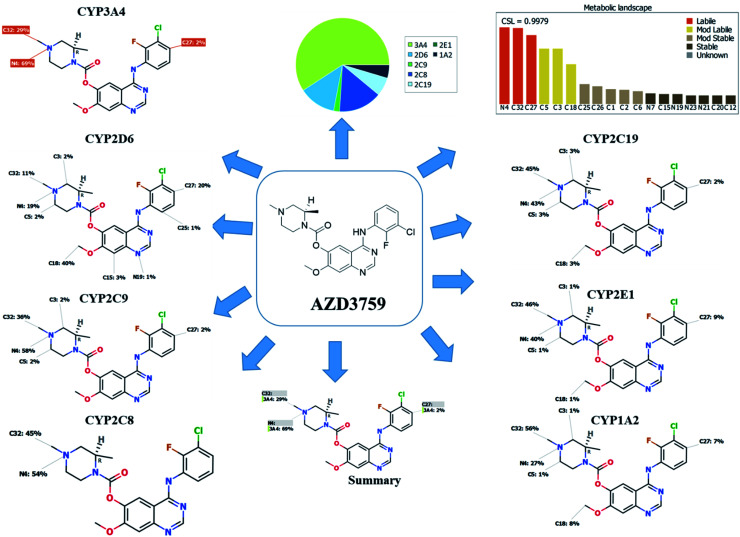
Predicted atomic sites of metabolism for zorifertinib by XenoSite web predictor.

The composite site lability for ZFB is 0.9979 that indicates the high lability of ZFB to metabolism. The metabolic Landscape indicates that atoms of C32 and N4 of *N*-methyl piperazine ring and C27 of phenyl group are the most labile atoms to metabolism, while C3 and C5 (the α carbons) of the *N*-methyl piperazine ring and C18 of methoxy group are moderate labile atoms to metabolism that matched with the current experiments (Fig. 1). Cyp3A4 had the main role in ZFB metabolism. Consecutively, depending on the literature knowledge and *in silico* postulations, a list of predicted reactive intermediates and metabolites was prepared and used as a guide during practical experiments.

### 3.2. *In silico* ZFB bioactivity and toxicity prediction

**Fig. 2:**
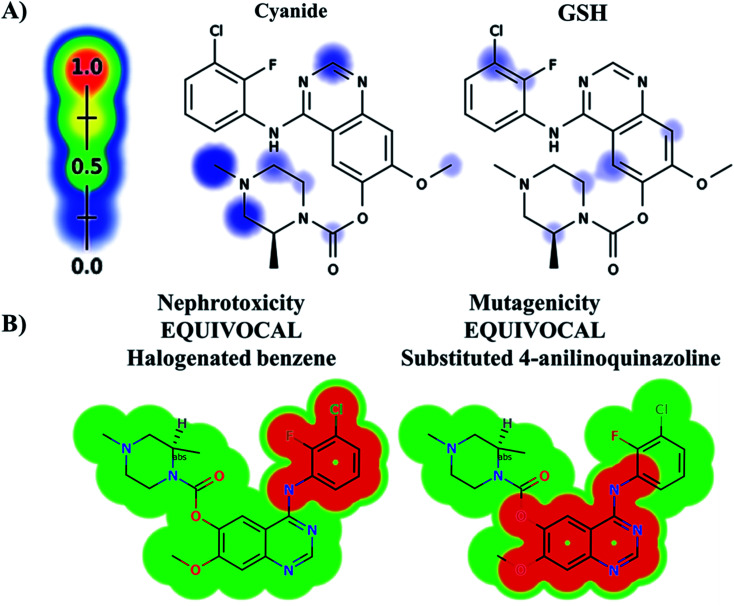
Predicted bioactive sites of zorifertinib by XenoSite web predictor including GSH and cyano bioactive centers (A), outcomes of *in silico* toxicity studies of zorifertinib using DEREK toxicity module of StarDrop software. Red color indicates structural alerts (B).

The ZFB atomic sites were proposed for generation of electrophiles that could be trapped by cyanide and GSH. The results were displayed as gradient color scale bar where the white color and red color indicates no or highest possibility of reactive intermediate formation at the atomic site, respectively (Fig. 2). GSH model reveals the possibility of reactive intermediate generation at phenyl group that could be trapped with GSH. KCN model reveals the possibility of reactive iminium ion intermediate generation at the piperazine ring that could be captured by cyanide forming cyano adduct that was confirmed by *in vitro* experiments. The outcomes of the predictions are shown in Fig. 2A.

*In silico* toxicity assessment of ZFB metabolites was done using DEREK module (StarDrop software package) (Fig. 2B). ZFB and its metabolites show equivocal nephrotoxicity and equivocal mutagenicity due to halogenated benzene and substituted 4-anilinoquinazoline, respectively (Fig. 2B). M4 shows plausible HERG channel inhibition due to 2-aminophenol derivative. Table 2 shows a complete list of *in vitro* and *in vivo* ZFB metabolites with DEREK outcomes for supposed toxicity profile.

**Table 2:** DEREK results for ZFB and its *in vitro* and *in vivo* metabolites

ZFB and its metabolites	Nephrotoxicity	Mutagenicity	HERG channel inhibition	Teratogenicity and other toxicological parameters
Structural alert	Halogenated benzene	Substituted 4-anilinoquinazoline	2-Aminophenol derivative	*para*-Phenylenediamine
AZD 3759	Equivocal	Equivocal	Na	Na
M1	Equivocal	Equivocal	Na	Na
M2	Equivocal	Equivocal	Na	Na
M3	Equivocal	Equivocal	Na	Na
M4	Equivocal	Equivocal	Plausible	Na
M5	Equivocal	Equivocal	Na	Na
M6	Equivocal	Equivocal	Na	Na
M7	Equivocal	Equivocal	Na	Na
M8	Equivocal	Equivocal	Na	Na
M9	Equivocal	Equivocal	Na	Na
M1	Equivocal	Equivocal	Na	Na
M10	Equivocal	Equivocal	Na	Na
M11	Equivocal	Equivocal	Na	Na
M12	Equivocal	Equivocal	Na	Na
M13	Equivocal	Equivocal	Na	Na
M14	Equivocal	Equivocal	Na	Na
M15	Equivocal	Equivocal	Na	Na

### 3.3. MS/MS fragmentation behavior of ZFB

**Fig. 3:**
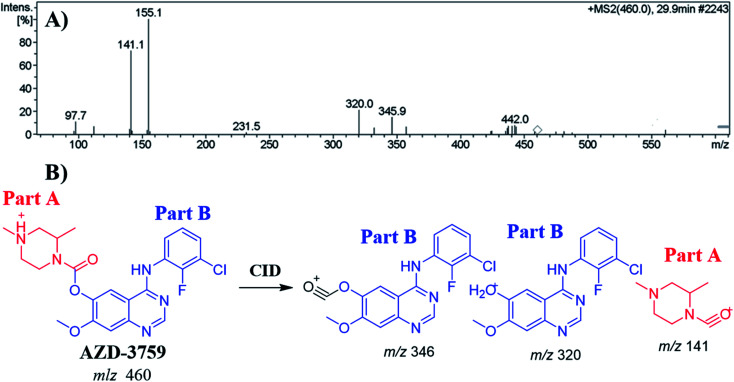
MS^2^ mass spectrum of zorifertinib (A). Proposed zorifertinib structure and its corresponding fragment ions (B).

The ZFB precursor ion peak (PIP) appeared at 29.9 min in the total ion chromatogram (TIC). Dissociation of precursor ion (PI) at *m*/*z* 460 resulted in three characteristic fragment ions at *m*/*z* 346, *m*/*z* 320 and *m*/*z* 141 (Fig. 3A). The dissociation of ZFB targets the weak carboxylate link between the piperazine and quinazoline moieties inside its chemical structure. Fragment ions at *m*/*z* 320 and *m*/*z* 141 represent the two building blocks of ZFB. Fragment ion at *m*/*z* 141 was used for confirmation of the metabolic reaction at piperazine ring while *m*/*z* 320 for the remaining chemical structure (Fig. 3B).^30^

### 3.4. Identification of ZFB *in vitro* metabolites

Purified extracts recovered from RLMs incubates were injected into LC-IT-MS. Six phase I metabolic reactions produced by *N*-demethylation, *O*-demethylation, hydroxylation, reduction, α-oxidation and dechlorination. One sulphate conjugate and two glucuronic acid conjugates were characterized after incubation of ZFB with isolated perfused RLHs as phase II metabolite (Table 3). *In vitro* phase I metabolites (M2, M3, M4, M5 and M6) figures and schemes have been moved to a ESI (Fig. S1–S5†). *In vitro* phase II metabolites (M9) figures and schemes have been moved to a ESI (Fig. S6†).

**Table 3:** *In vitro* phase I and metabolites of ZFB

	MS scan	Major fragment ions	*t*_R_ (min)	Proposed metabolic reaction
ZFB	460	320, 285, 127, 141	29.9	ZFB

***In vitro* phase I metabolites**				
M1	446	320, 428, 285, 100	27.6	*N*-Demethylation at piperazine ring
M2	446	306, 428	45.3	*O*-Demethylation
M3	476	320, 458	27.7	Hydroxylation
M4	434	306, 416, 287	34.7	*N*-Demethylation, *O*-demethylation and reduction
M5	456	438, 173	36.8	α-Oxidation, dechlorination, defluorination then hydroxylation
M6	430	412, 335, 161	44.2	*N*-Demethylation, *O*-demethylation, hydroxylation, and dechlorination

***In vitro* phase I metabolites**				
Sulphate conjugates				
M7	526	446, 508	44.1	*N*-Demethylation and conjugation of sulphate group
Glucuronide conjugates				
M8	622	430, 446, 605	26.7	*O*-Demethylation, and glucuronide conjugates
M9	648	472, 438, 630	29.1	Hydroxylation, α-oxidation, dechlorination then hydroxylation and, glucuronide conjugates

#### 3.4.1. Identification of the M1 metabolite of ZFB

**Fig. 4:**
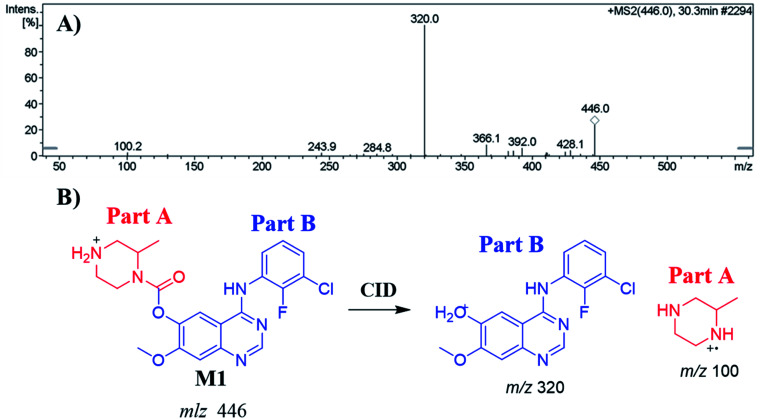
MS^2^ mass spectrum of M1 (A). Proposed M1 structure and its corresponding fragment ions (B).

The M1 PIP appeared at 30.3 min in the TIC. Dissociation of PI at *m*/*z* 446 resulted in two characteristic fragment ions at *m*/*z* 320 and *m*/*z* 100 (Fig. 4A). Comparing to the dissociation of ZFB, fragment ion at *m*/*z* 320 indicates that no metabolic reaction in part B while part A shows a 14 *m*/*z* less that indicates the *N*-demethylation of piperazine ring (Fig. 4B). Radical cation (*m*/*z* 100) was formed in small intensity due to collision induced dissociation (CID) in LC-IT-MS.^31^

#### 3.4.2. Identification of the M7 metabolite of ZFB

**Fig. 5:**
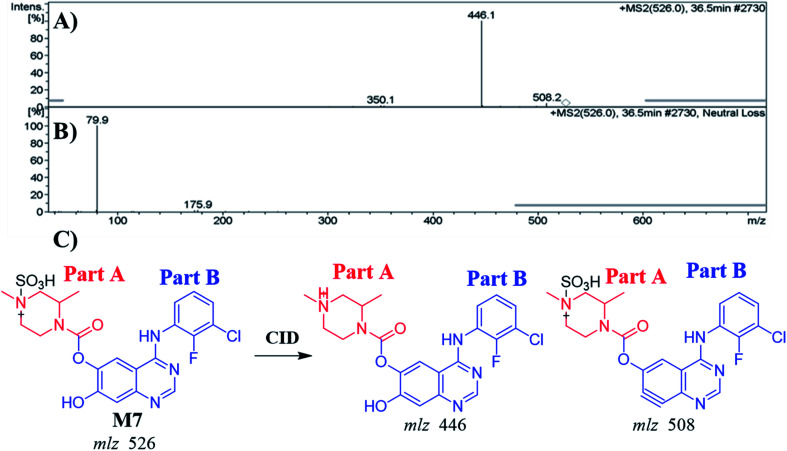
MS^2^ mass spectrum of M7 (A). Constant neutral loss scan of M7 (B). Proposed M7 structure and its corresponding fragment ions (C).

The M7 PIP appeared at 36.5 min (*m*/*z* 526) in the TIC. PIP exhibits 80 *m*/*z* higher than that of the M1 (precursor metabolite) with *m*/*z* 446 that was predicted to be sulfate conjugation of M1. Dissociation of PI at *m*/*z* 526 resulted in two characteristic fragment ions at *m*/*z* 508 and *m*/*z* 446 (Fig. 5A). Comparing to the dissociation of ZFB,^32^ fragment ion at *m*/*z* 446 indicates that the metabolic reaction in part B (14 *m*/*z* less) while part A shows no metabolic reaction that indicates the *O*-demethylation of methoxy group attached to quinazoline moiety. Fragment ion at *m*/*z* 508 (18 *m*/*z* less) that indicates water loss and confirm the *O*-demethylation metabolic reaction. M7 was proposed to be a direct conjugation of M1 with sulfate ion (Fig. 5B). Another LC-IT-MS screening for sulfate adduct was done by neutral loss scan of ions that lose 80 Da (Fig. 5C).^33^

#### 3.4.3. Identification of the M8 metabolite of ZFB

**Fig. 6:**
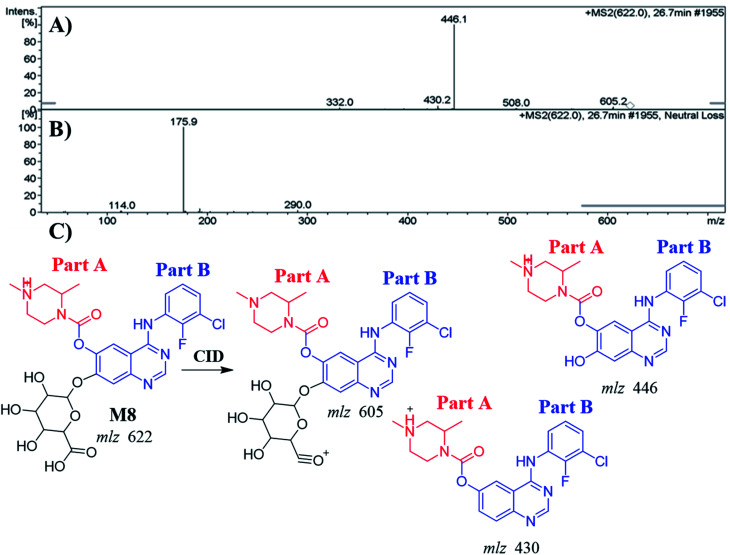
MS^2^ mass spectrum of M8 (A). Constant neutral loss scan of M8 (B). Proposed M8 structure and its corresponding fragment ions (C).

The M8 PPI appeared at 26.7 min in the TIC. Dissociation of PI at *m*/*z* 622 resulted in three characteristic fragment ions at *m*/*z* 605, *m*/*z* 446 and *m*/*z* 430 (Fig. 6A). It showed 176 *m*/*z* higher than that of PI with *m*/*z* 446 (M2), which proposed to be a glucuronic acid conjugate of ZFB metabolite. Fragment ion at *m*/*z* 446 that showed neutral loss of glucuronic acid (loss of 176 *m*/*z* units) that matched with the other fragment ions at *m*/*z* 430 and *m*/*z* 605 (Fig. 6B and C). Constant neutral loss scan for M8 metabolite conjugates are more specific (due to the characteristic loss of 176 mass units), and provide much cleaner mass spectral data.^32^

### 3.5. Identification of ZFB *in vivo* metabolites

PIP mass spectra comparison between urine extracts with control urine samples as well as PIP comparison of ZFB proposed metabolites (Table 4) allowed the profiling of six *in vivo* phase I and three *in vivo* phase II metabolites. Metabolic pathways for *in vivo* phase I metabolites were supposed to be *N*-demethylation, hydroxylation, reduction, dechlorination and α oxidation, while for phase II metabolites were the result of two glucuronic acid conjugation and one sulphate (Table 4). M1 metabolites is previously mentioned in *in vitro* ZFB phase I metabolism. M7 and M9 metabolites are previously mentioned in *in vivo* ZFB phase II metabolism. *In vivo* phase I metabolites (M10, M11, M12, M13 and M14) figures and schemes have been moved to a ESI (Fig. S7–S10†).

**Table 4:** *In vivo* phase I and phase II metabolites of ZFB using MS scan and fragment ions

	MS scan	Major fragment ions	*t*_R_ (min)	Proposed metabolic reaction
***In vivo* phase I metabolites**				
M1	446	320, 285, 428, 100	30.3	*N*-Demethylation at piperazine ring
M10	476	336, 141	26.4	Hydroxylation
M11	476	320, 458	34.3	Hydroxylation
M12	476	320, 458	35.4	Hydroxylation
M13	462	320, 143, 443	37.1	Reduction
M14	488	320, 169	38.9	α Oxidation

***In vivo* phase II metabolites**				
Sulphate conjugate				
M7	526	446, 508	36.5	*N*-Demethylation and conjugation of sulphate group
Glucuronide conjugates				
M9	648	472, 438, 630	29.1	Hydroxylation, α oxidation, dechlorination then hydroxylation and, glucuronide conjugates
M15	638	462, 141	26.8	Hydroxylation, *O*-demethylation of benzene group and conjugation of glucuronic acid

#### 3.5.1. Identification of the M15 metabolite of ZFB

**Fig. 7:**
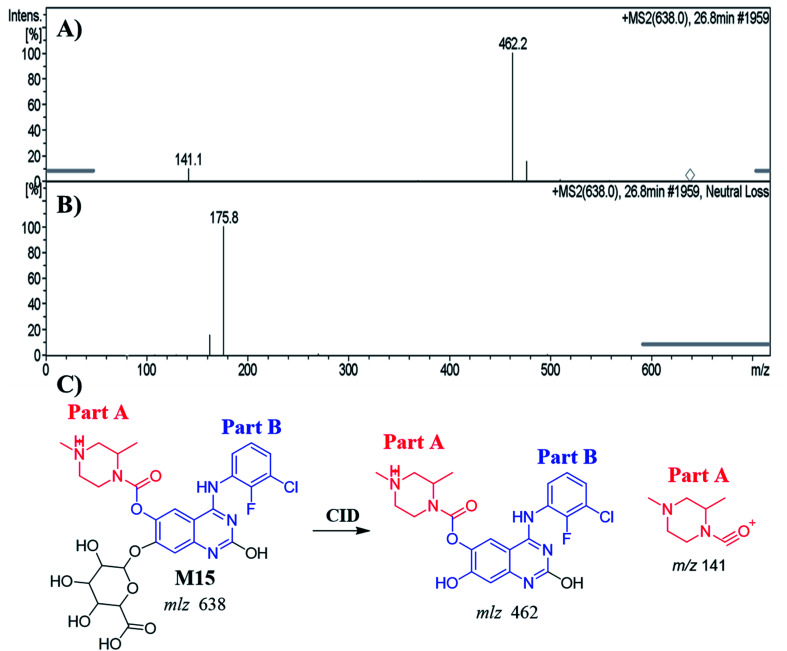
MS^2^ mass spectrum of M15 (A). Constant neutral loss scan of M15 (B). Proposed M15 structure and its corresponding fragment ions (C).

The M15 PIP appeared at 26.8 min in the TIC. Dissociation of PI at *m*/*z* 638 resulted in two characteristic fragment ions at *m*/*z* 462 and *m*/*z* 141 (Fig. 7A). Comparing to the dissociation of ZFB, fragment ion at *m*/*z* 141 indicates that no metabolic reaction in part A. Fragment ion at 462 indicates the neutral loss of glucuronic acid (loss of 176 *m*/*z*) that indicates *O*-glucuronidation after *O*-demethylation of methoxy group. The proposed metabolic reactions were hydroxylation reaction at quinazoline moiety, *O*-demethylation and *O*-glucuronidation (Fig. 7B and C). Glucuronidation was confirmed by running neutral loss scan mode that shows the precursor ion at *m*/*z* 638.^32^

### 3.6. Identification of *in vitro* reactive metabolites of ZFB

The same metabolic incubation (ZFB with RLMs) was done in the presence of 1.0 mM KCN and 1.0 mM GSH to trap iminium and electro deficient conjugated system intermediates, respectively. Four GSH conjugates and three cyanide adducts were identified (Table 5), revealing that the *N*-phenyl amine group and methyl piperazine ring in ZFB can become bioactivated and then trapped by the nucleophile GSH and cyanide ion.^34^ ZFB cyano adducts (AZDCN515, AZDCN501) and ZFB GSH conjugates (AZDGSH761, AZDGSH763, AZDGSH717) figures and schemes have been moved to a ESI (Fig. S11–S15†).

**Table 5:** Reactive metabolites of ZFB

	MS scan	Most abundant fragment ions	Rt. (min)	Metabolic reaction
**Cyanide adducts**				
AZDCN485	485	148, 458	46.6	Attack of KCN at bioactivated piperazine ring of parent drug
AZDCN515	515	371, 488, 197	43.2	Hydroxylation, α-oxidation and attack of KCN at bioactivated piperazine ring
AZDCN501	501	294, 327, 474	45.5	Hydroxylation at piperazine ring and attack of KCN at bioactivated piperazine ring

**GSH conjugates**				
AZDGSH715	715	586, 398	42.4	*N*-Demethylation, dechlorination, defluorination then hydroxylation and GSH conjugate at benzene ring
AZDGSH761	761	632, 731	43.1	Double hydroxylation at piperazine ring, dechlorination, defluorination then hydroxylation and conjugation of GSH at benzene ring
AZDGSH763	763	634, 458	36.4	*O*-Demethylation, double hydroxylation at piperazine ring, dechlorination, defluorination then hydroxylation and conjugation of GSH at benzene ring
AZDGSH717	717	588, 307	31	*N*-Demethylation, reduction, dechlorination, defluorination then hydroxylation and conjugation of GSH at benzene ring

#### 3.6.1. Identification of AZDCN485 cyano adduct

**Fig. 8:**
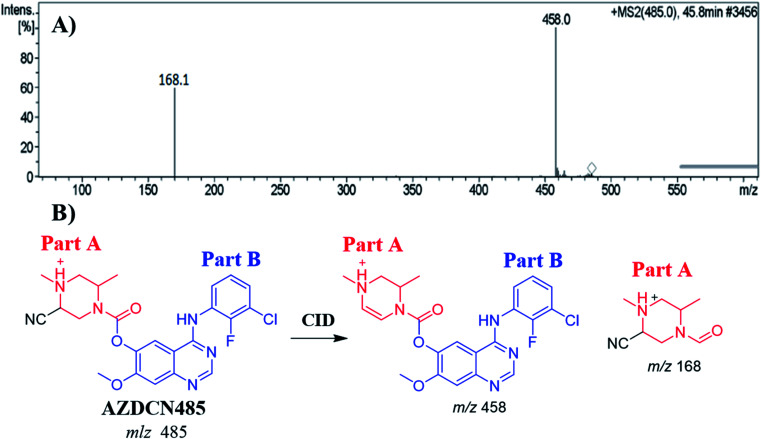
MS^2^ mass spectrum of AZDCN485 (A). Proposed AZDCN485 structure and its corresponding fragment ions (B).

The AZDCN485 PIP appeared at 46.6 min in the total ion chromatogram (TIC). Dissociation of PI at *m*/*z* 485 resulted in two characteristic fragment ions at *m*/*z* 458 and *m*/*z* 168 (Fig. 8A). Fragment ion at *m*/*z* 458 (27 *m*/*z* less) that indicates HCN loss and confirms the cyano adduct formation. Comparing to the dissociation of ZFB, fragment ion at *m*/*z* 168 indicates that cyano nucleophile trapped the bioactivated piperazine ring (part A) forming cyano adduct (Fig. 8B).

#### 3.6.2. Identification of AZDGSH715 GSH conjugates

**Fig. 9:**
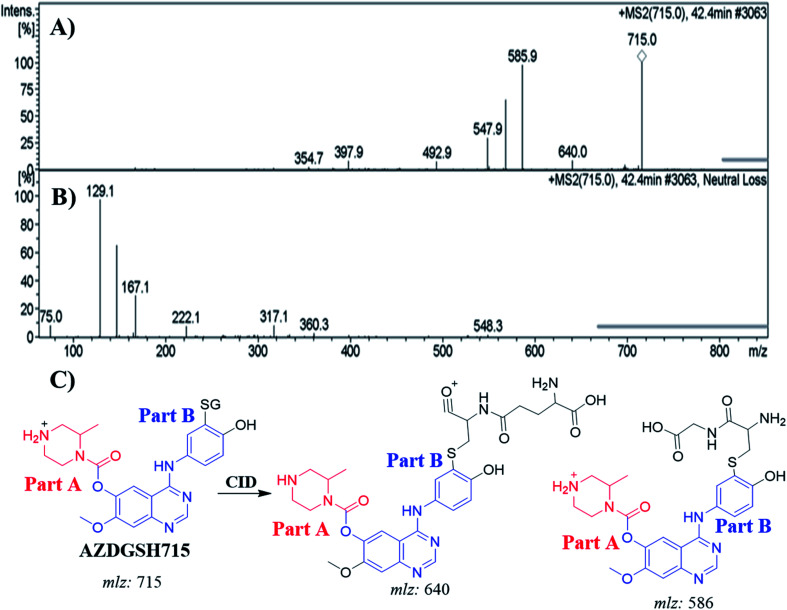
MS^2^ mass spectrum of AZDGSH715 (A). Constant neutral loss scan of AZDGSH715 (B). Proposed AZDGSH715 structure and its corresponding fragment ions (C).

The AZDGSH715 PIP appeared at 42.4 min in the TIC. Dissociation of PI at *m*/*z* 715 resulted in two characteristic fragment ions at *m*/*z* 640 and *m*/*z* 586 (Fig. 9A). Fragment ion at *m*/*z* 586 (129 *m*/*z* less) that indicates anhydroglutamic acid moiety and confirms the formation of GSH conjugate. Constant neutral loss scan for screening for GSH adduct was done by monitoring of ions that lose 129 Da (Fig. 9B).^32^ AZDGSH715 formation indicated the phenyl ring bioactivation in *in vitro* metabolism of ZFB. The metabolic reactions that occurred in AZDGSH715 were proposed dechlorination, *N*-demethylation, defluorination then hydroxylation and GSH conjugation at bioactivated phenyl ring (iminoquinone derivative) (Fig. 9C).

### 3.7. Proposed pathways of bioactivation of ZFB

**Fig. 10:**
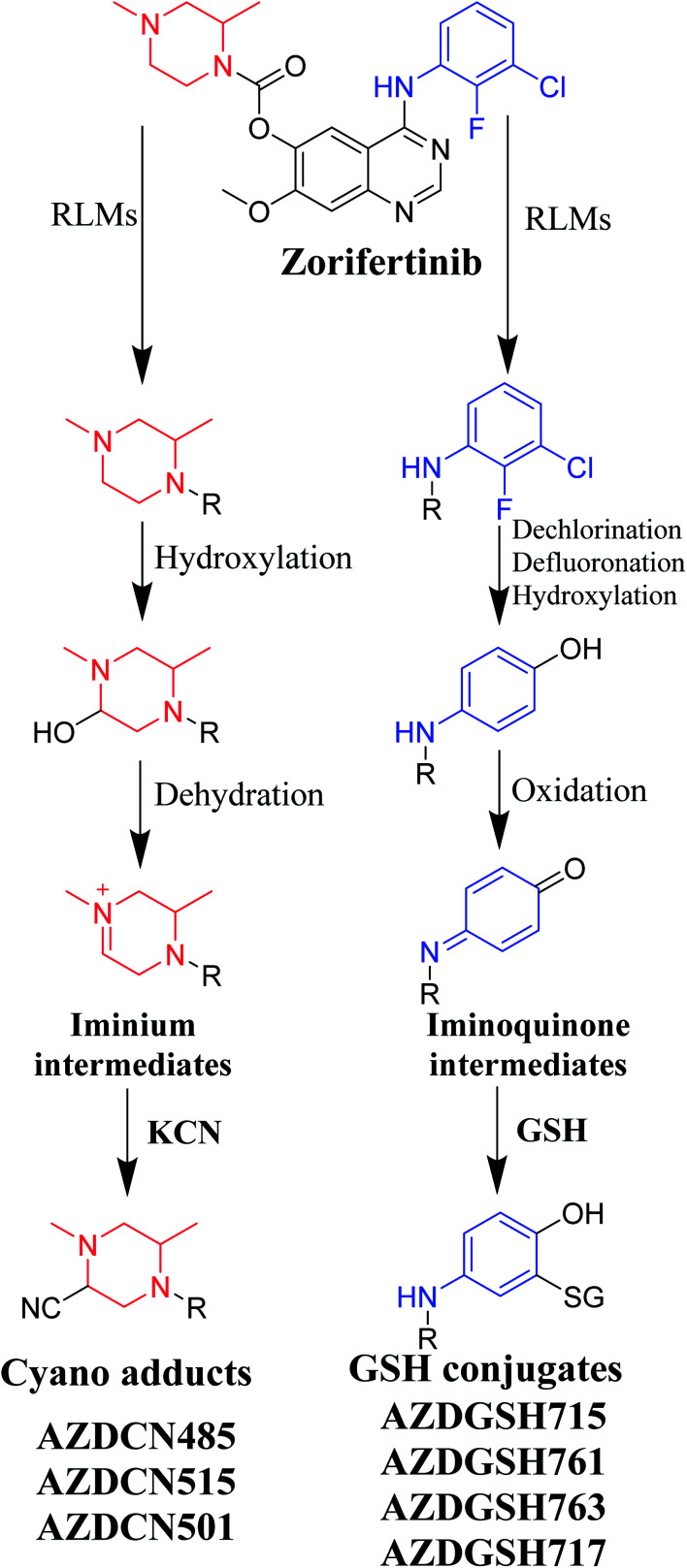
Proposed pathways for zorifertinib bioactivation for cyano adducts and GSH conjugates.

Bioactivation pathways of ZFB are proposed in Fig. 10. AZDCN485, AZDCN515 and AZDCN501 cyanide adducts revealed the metabolic formation of reactive iminium intermediates at *N*-methyl piperazine ring in ZFB metabolism. The bioactivation pathway was predicted as hydroxylation metabolic reaction at *N*-methyl piperazine ring in ZFB followed by loss of H_2_O (dehydration) that resulted in iminium ions intermediates (reactive hard nucleophile) that were trapped using cyanide electrophile (KCN) forming stable cyanide adducts that were characterized and identified using LC-IT-MS. This bioactivation pathway that involves iminium intermediates formation is previously reported with other drugs containing cyclic tertiary amine ring.^35,36^ The formation of AZDGSH715, AZDGSH761, AZDGSH763 and AZDGSH717 supposed the formation of iminoquinone reactive intermediates in the metabolism of ZFB that were formed by dechlorination, defluorination and hydroxylation of phenyl group and then oxidation^34^ generating reactive intermediates that were trapped using GSH leading to the formation of stable conjugates that were characterized and identified using LC-IT-MS (Fig. 10).^37^

## 4. Conclusions

**Fig. 11:**
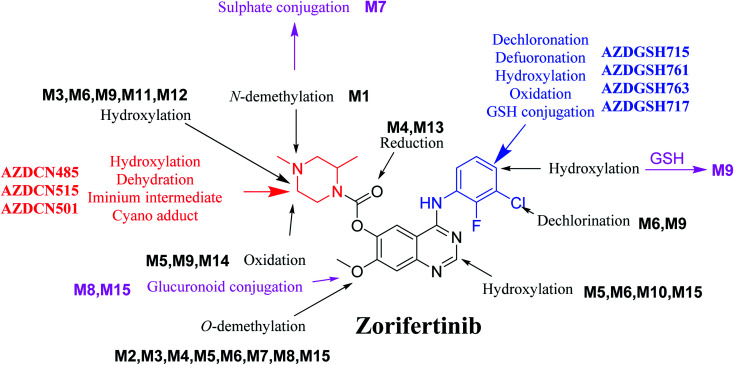
Chemical structure of zorifertinib showing bioactivation pathways including iminium, and electro deficient conjugated system.

*In silico* software (WhichP450™ module, DEREK and XenoSite reactivity model) has proposed the most labile atoms in the ZFB structure that revealed to be the same as seen in the detected metabolites in phase I metabolism and the same groups were bioactivated as expected. Three *in vitro* phase II metabolites (sulphate and glucuronic acid conjugates) and six *in vitro* phase I (*N*-demethylation, *O*-demethylation, hydroxylation, reduction, and oxidation) and were found for ZFB. Three *in vivo* phase II metabolites (one sulphate and two glucuronic acid conjugates) and six *in vivo* phase I metabolites (*N*-demethylation, α-oxidation, hydroxylation, reduction, and dechlorination) of ZFB were identified (Fig. 11). Seven reactive intermediates (four GSH conjugates and three cyano adducts) were characterized and the mechanisms of their formation were proposed (Fig. 11) that may illuminate the way for figuring out the causes behind ZFB toxic side effects.

## Ethics approval

Ethical approval for the Animal experiments of were obtained from the Animal Ethics Committee of King Saud University (No. KSU-SE-19-52).

## Author contributions

Nasser S. Al-Shakliah: conceptualization, methodology, visualization, software. Mohamed Attwa: data curation, writing – original draft preparation. Adnan Kadi: supervision, investigation, software, validation. Haitham AlRabiah: supervision, writing – reviewing and editing. Haya Aljohar: writing – reviewing and editing.

## Conflicts of interest

The authors declare no conflicts of interest for the current work.

## Abbreviations

LC-IT-MS: Liquid chromatography ion trap mass spectrometry
ACN: Acetonitrile
ESI: Electrospray ionization
RLMs: Rat liver microsomes
TKIs: Tyrosine kinase inhibitors
PIP: Precursor ion peak
TIC: Total ion chromatogram
CID: Collision-induced dissociation

## Supplementary Material

RA-012-D2RA02848D-s001
