# Supplementary material for: Profiling of in vivo, in vitro and reactive zorifertinib metabolites using liquid chromatography ion trap mass spectrometry
Source: RSC Adv. 2022 Jul 21;12(32):20991–1003. doi: 10.1039/d2ra02848d (PMC9301632; doi:10.1039/d2ra02848d)
Supplement: RA-012-D2RA02848D-s001 [file RA-012-D2RA02848D-s001.pdf]

Electronic Supplementary Material (ESI) for RSC Advances.
This journal is © The Royal Society of Chemistry 2022

Supplementary data

1. *In vitro* phase I metabolites

1.1. Identification of the M2 metabolite of ZFB

The M2 PIP appeared at 45.3 min in the TIC. Fragmentation of PI at *m/z* 446 resulted in two characteristic fragment ions at *m/z* 428 and *m/z* 306 (Fig. S1A). Comparing to the fragmentation of ZFB, fragment ion at *m/z* 306 indicates that the metabolic change in part B (14 *m/z* less) while part A shows no metabolic change that indicates the *O*-demethylation of methoxy group attached to quinazoline moiety. Fragment ion at m/z 428 (18 *m/z* less) that indicates water loss and confirm the *O*-demethylation metabolic change (Fig. S1B).

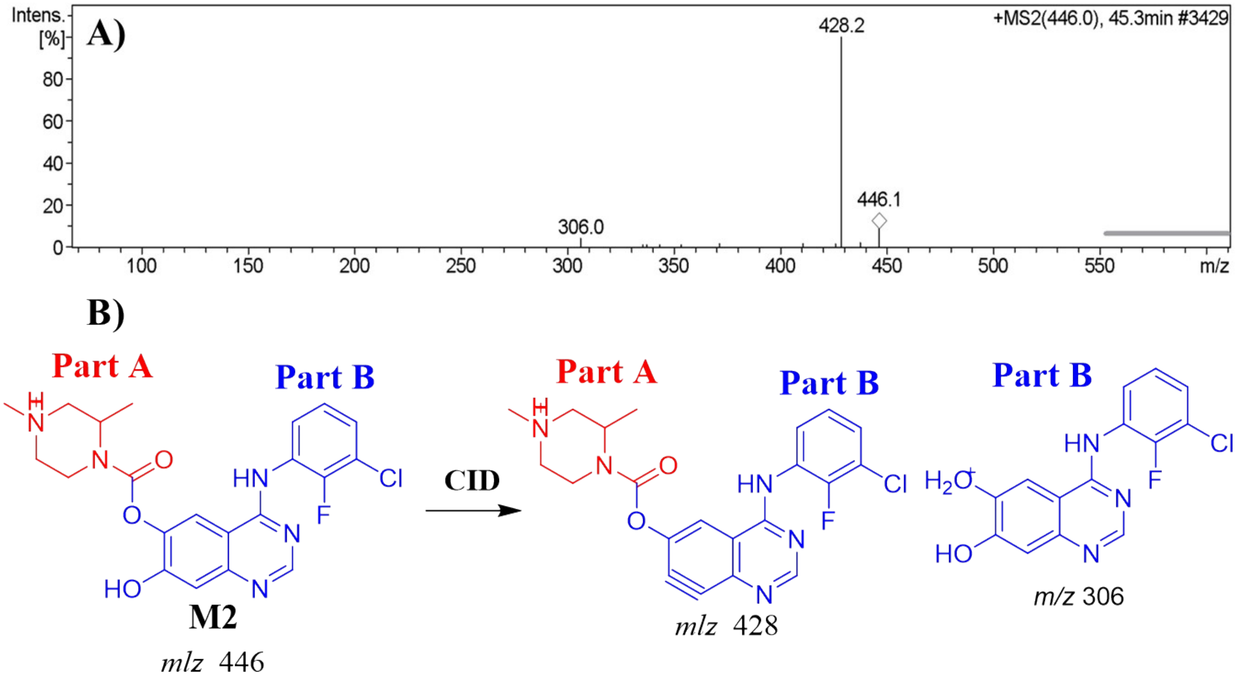

Fig. S1. MS^2 mass spectrum of M2 (A). Proposed M2 structure of and corresponding daughter ions (B).

1.2. Identification of the M3 metabolite of ZFB

The M3 PIP appeared at 27.7 min. in the TIC. Fragmentation of PI at *m/z* 476 resulted in two characteristic fragment ions at *m/z* 458 and *m/z* 320 (Fig. S2A). Comparing to the fragmentation of ZFB, fragment ion at *m/z* 320 indicates that no metabolic change in part B while part A shows hydroxylation metabolic change that indicates the hydroxylation at

piperazine ring. Fragment ion at m/z 458 (18 *m/z* less) that indicates water loss and confirm the hydroxylation metabolic pathway (Fig. S2B).

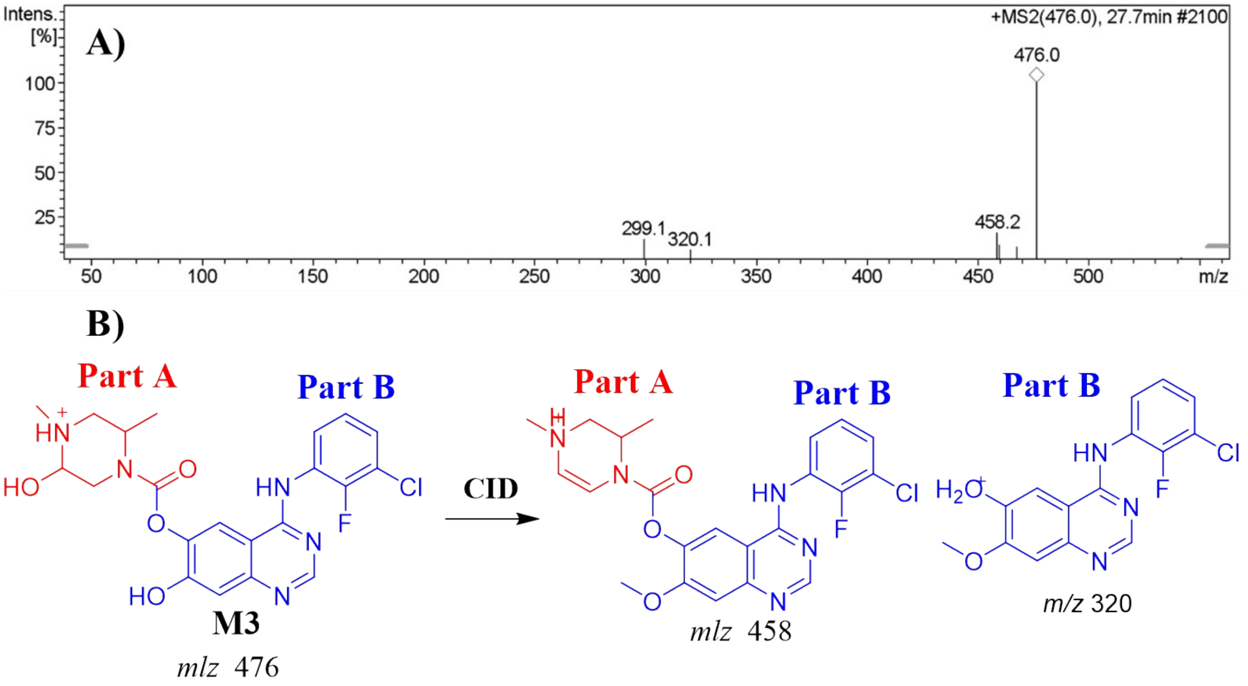

Fig. S2. MS^2 mass spectrum of M3 (A). Proposed M3 structure and its corresponding daughter ions (B).

1.3. Identification of the M4 metabolite of ZFB

The M4 PIP appeared at 34.6 min. in the TIC. Fragmentation of PI at *m/z* 434 resulted in three characteristic fragment ions at *m/z* 458, *m/z* 305 and *m/z* 287 (Fig. S3A). Comparing to the fragmentation of ZFB, fragment ion at *m/z* 305 indicates that *O*-demethylation metabolic change at part B and *N*-demethylation and reduction metabolic reaction of carbonyl group at part A. Fragment ion at m/z 287 (18 *m/z* less) that indicates water loss and confirm the *O*-demethylation metabolic pathway (Fig. S3B).

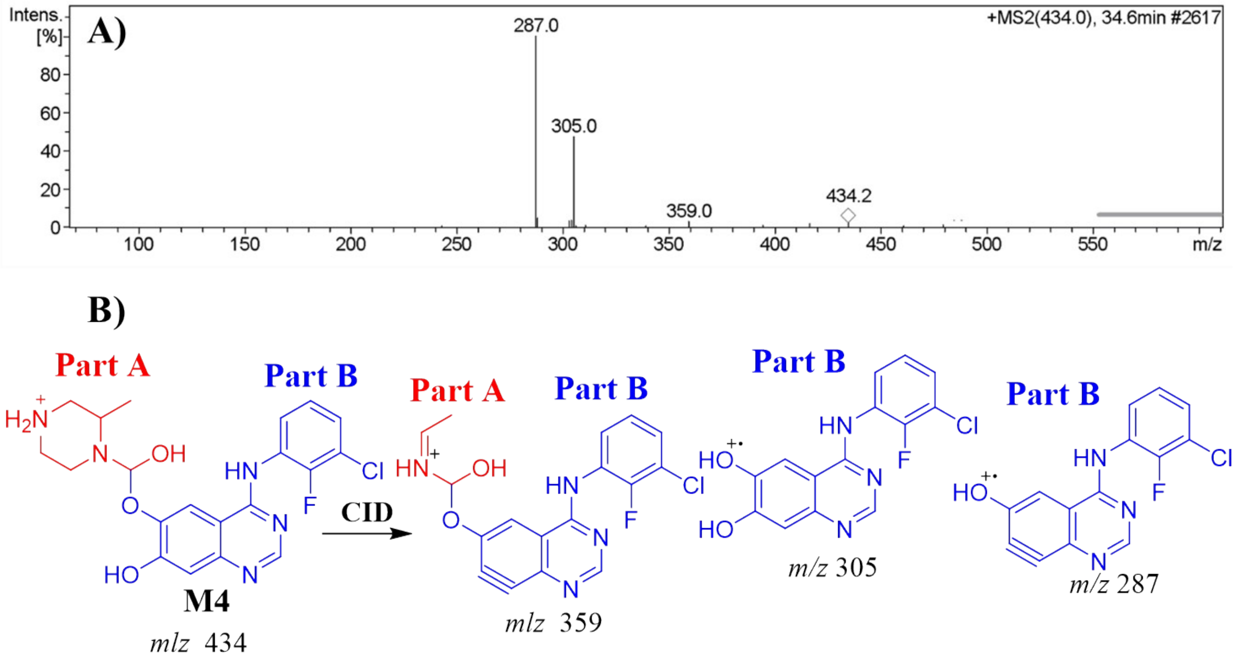

Fig. S3. MS2 mass spectrum of M4 (A). Proposed M4 structure and its corresponding daughter ions (B).

1.4. Identification of the M5 metabolite of ZFB

The M5 PIP appeared at 36.8 min. in the TIC. Fragmentation of PI at *m/z* 456 resulted in two characteristic fragment ions at *m/z* 438 and *m/z* 173 (Fig. S4A). Comparing to the fragmentation of ZFB, fragment ions at *m/z* 438 and 173 indicates α- oxidation at the piperazine moiety of ZFB and dechlorination, defluorination then double hydroxylation metabolic reactions at phenyl ring of ZFB. (Fig. S4B).

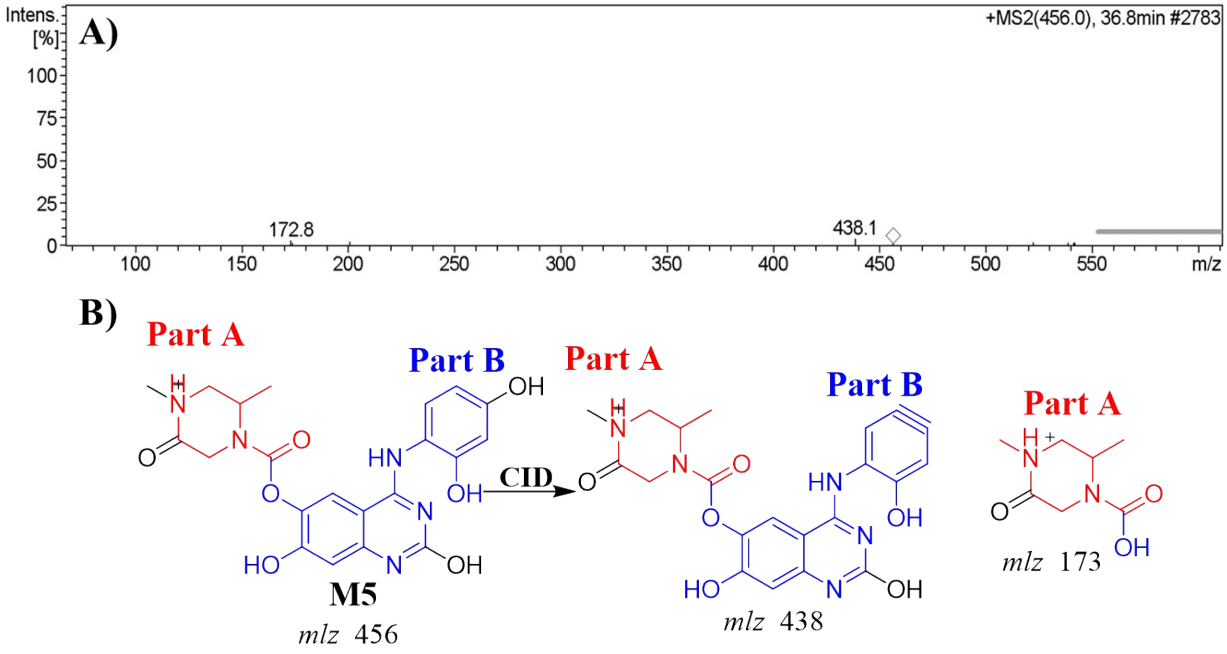

Fig. S4. MS^2 mass spectrum of M5 (A). Proposed M5 structure and its corresponding daughter ions (B).

1.5. Identification of the M6 metabolite of ZFB

The M6 PIP appeared at 44.2 min. in the TIC. Fragmentation of PI at *m/z* 430 resulted in three characteristic fragment ions at *m/z* 412, *m/z* 335 and *m/z* 161 (Fig. S5A). Comparing to the fragmentation of ZFB, fragment ion at *m/z* 412 indicates the loss of two methyl groups from piperazine and quinazoline rings of ZFB and the addition of two hydroxyl groups at piperazine and quinazoline rings of ZFB which matched with the other fragment ions at m/z 335 and m/z 161 (Fig. S5B).

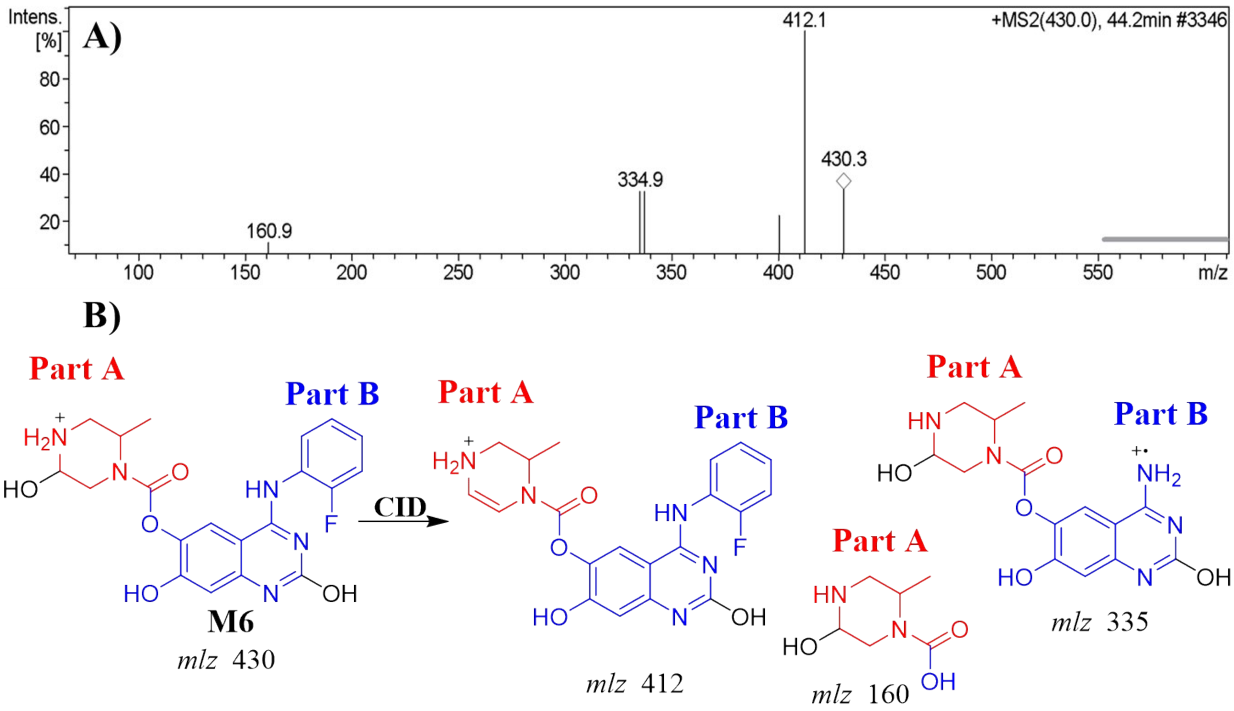

Fig. S5. MS^2 mass spectrum of M6 (A). Proposed M6 structure and its corresponding daughter ion (B).

2. *In vitro phase II metabolites*

2.1. Identification of the M9 metabolite of ZFB

The M9 PPI appeared at 29.1 min. in the TIC. Fragmentation of PI at *m/z* 648 resulted in three characteristic fragment ions at *m/z* 630, *m/z* 472 and *m/z* 438 (Fig.S6A). It showed 176 m/z higher than that of PI with m/z 472 that proposed to be a glucuronic acid conjugate of ZFB metabolite. Fragment ion at *m/z* 472 showed a neutral loss of glucuronic acid (loss of 176 m/z units) that matched with the other fragment ions at m/z 630 and m/z 438 (Fig. S6B and S6C). Constant neutral loss scan for M9 metabolite conjugates are more specific (due to the characteristic loss of 176 mass units), and provide much cleaner mass spectral data.

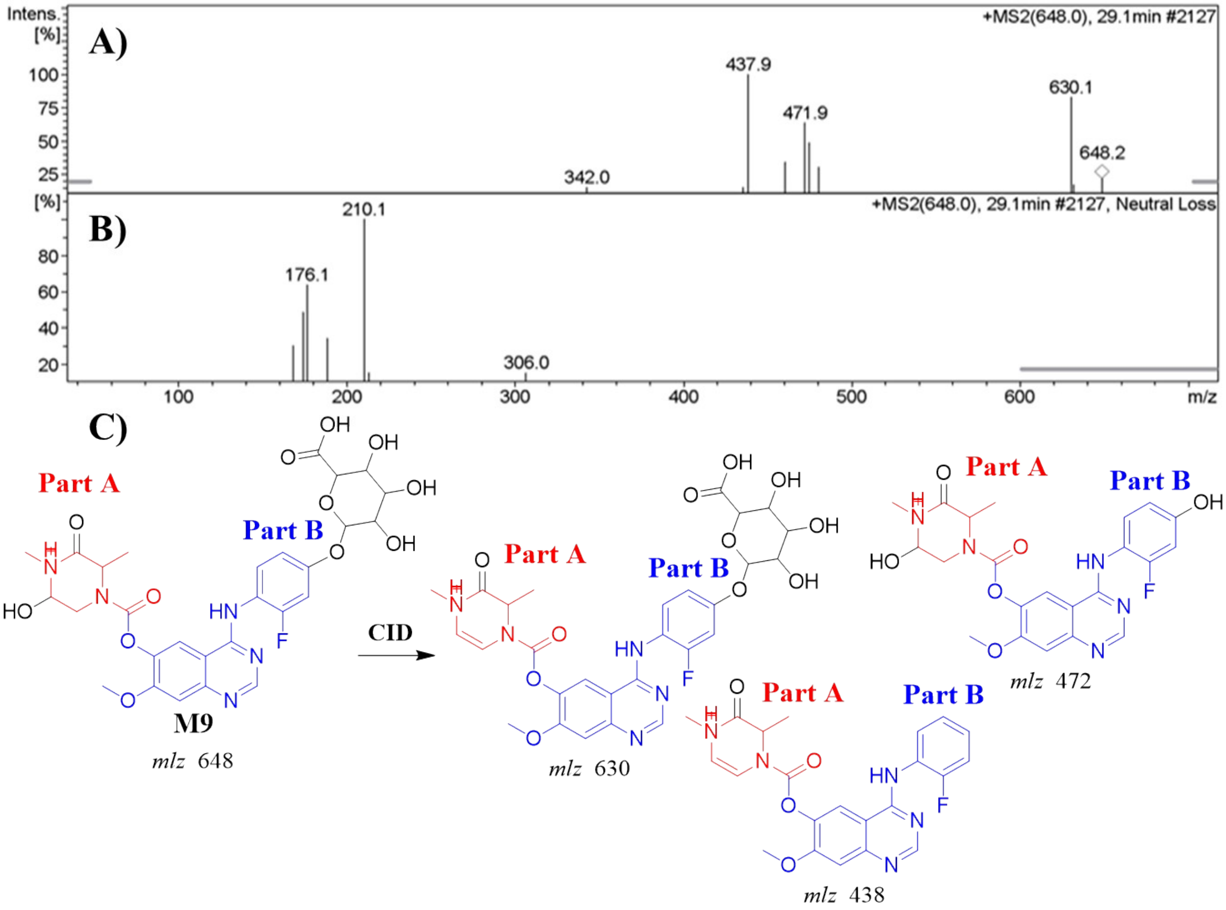

Fig. S6. MS^2 mass spectrum of M9 (A). Constant neutral loss scan of M9 (B). Proposed M9 structure and its corresponding daughter ions (C).

3. *In vivo phase I metabolites*

3.1. Identification of the M10 metabolite of ZFB

The M10 PIP appeared at 26.4 min in the TIC. Fragmentation of PI at *m/z* 476 resulted in two characteristic fragment ions at *m/z* 336 and *m/z* 141 (Fig. S7A). Comparing to the fragmentation of ZFB, fragment ion at *m/z* 336 indicates that the metabolic change in part B (16 *m/z* more) while part A shows no metabolic change that indicates the hydroxylation reaction at quinazoline moiety that matched with the fragment ion at m/z 141 (Fig. S7B).

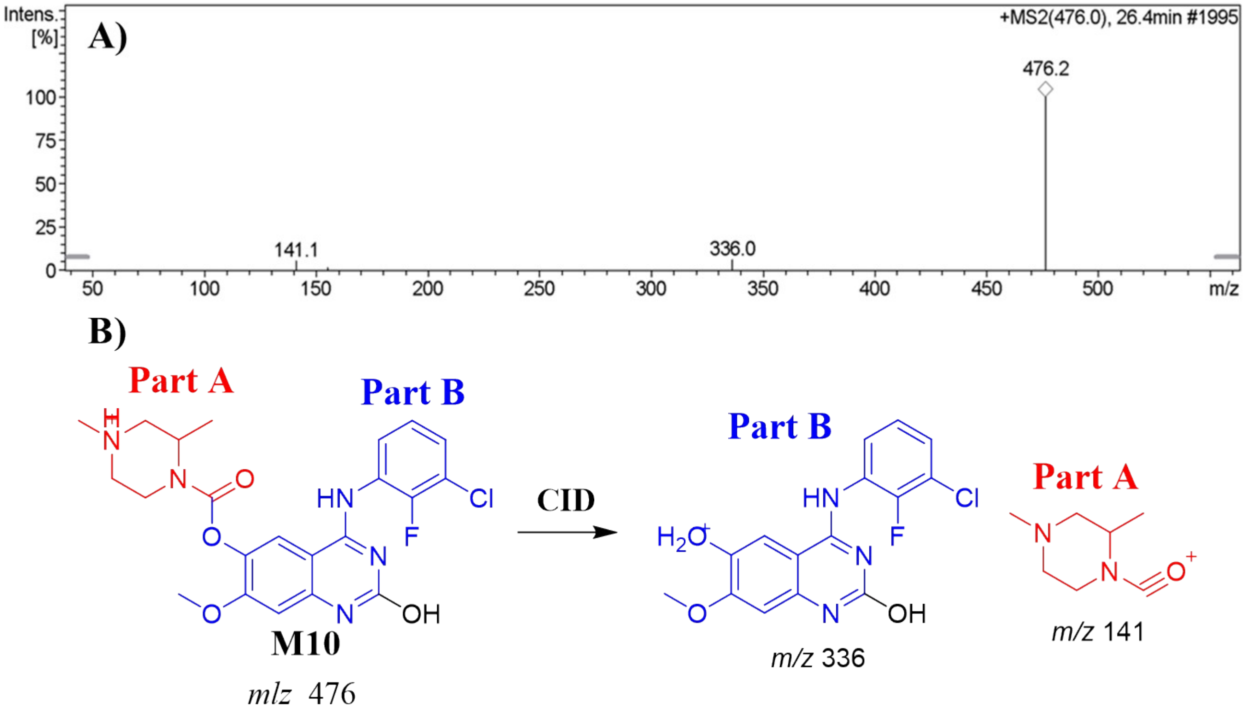

Fig. S7. MS^2 mass spectrum of M10 (A). Proposed M10 structure and its corresponding daughter ions (B).

3.2. Identification of the M11& M12 metabolite of ZFB

The M11 and M12 PIP appeared at 34.3 min. and 35.4 min. respectively in the TIC. Fragmentation of PIs at *m/z* 476 resulted in two characteristic daughter ions at *m/z* 458 and *m/z* 320 (Fig. S8A and S8B). Comparing to the fragmentation of ZFB, fragment ion at *m/z* 320 indicates that no metabolic change in part B while part A shows metabolic change that indicates the hydroxylation reaction at piperazine moiety. The fragment ion at m/z 458 (water loss) indicates hydroxylation metabolic reaction. M11 and M12 metabolites were

the net product of hydroxylation at two different positions at the piperazine ring of ZFB (Fig. S8C).

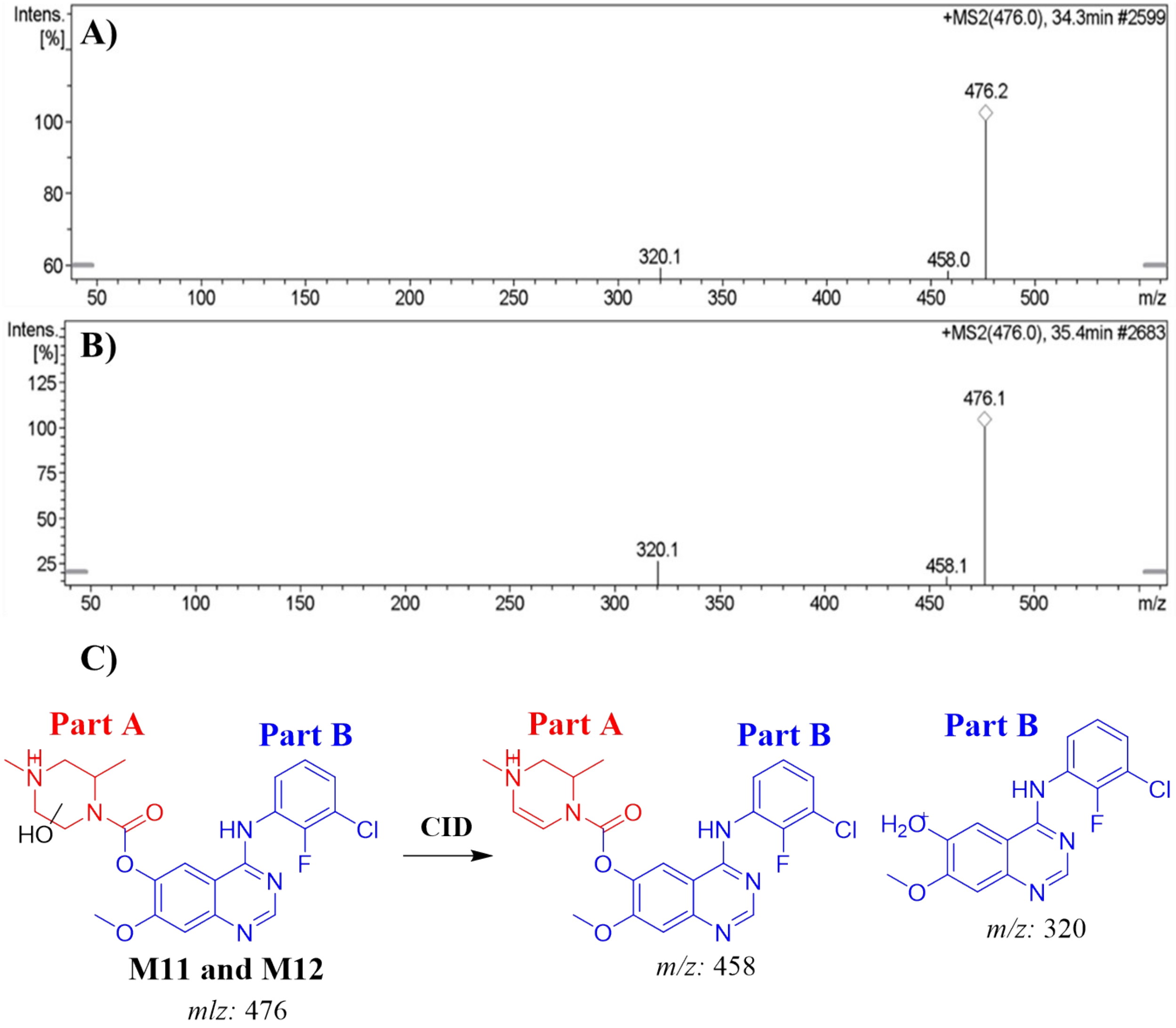

Fig. S8. MS^2 mass spectrum of M11 (A). MS2 mass spectrum of M12 (B). Proposed M11 and M12 structures and their corresponding daughter ions (C).

3.3. Identification of the M13 metabolite of ZFB

The M13 PIP appeared at 37.1 min in the TIC. Fragmentation of PI at *m/z* 462 resulted in three characteristic fragment ions at *m/z* 443, *m/z* 320 and *m/z* 143 (Fig. S9A). Comparing to the fragmentation of ZFB, fragment ion at *m/z* 336 indicates that the metabolic change in part B (2 *m/z* more) while part B shows no metabolic change that indicates the reduction metabolic reaction at carbonyl group (Fig. S9B).

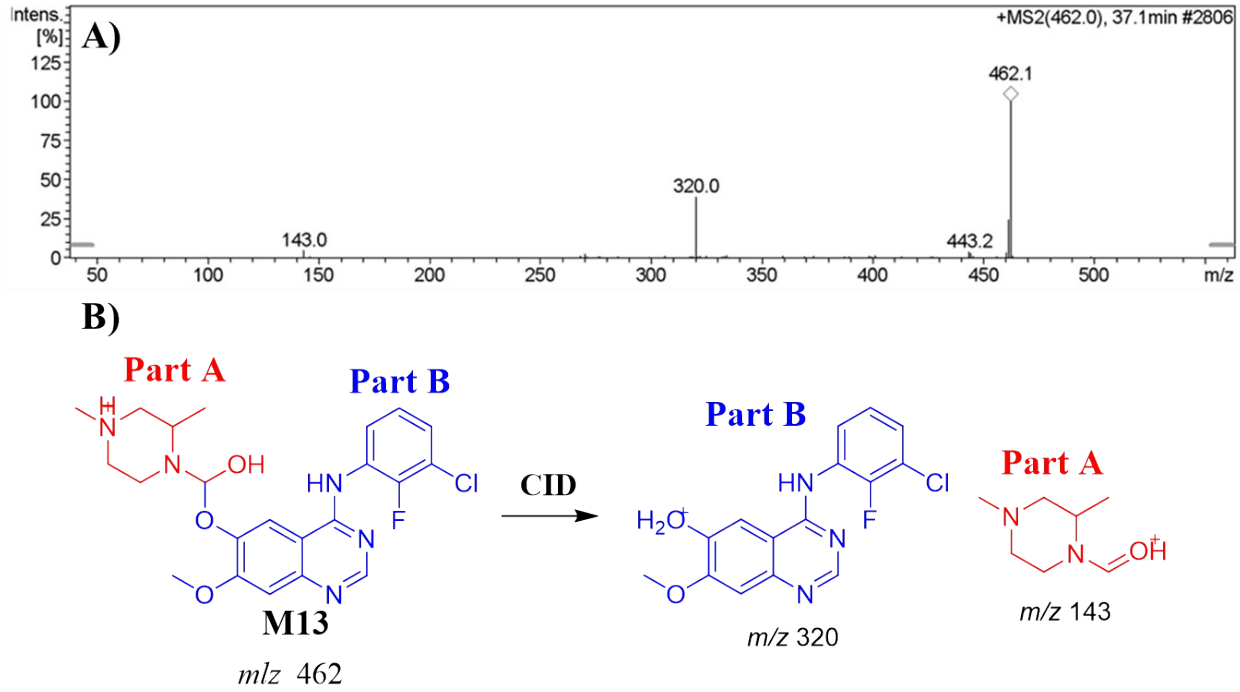

Fig. S9. MS^2 mass spectrum of M13 (A). Proposed M13 structure and its corresponding daughter ions (B).

3.4. Identification of the M14 metabolite of ZFB

The M14 PIP appeared at 38.9 min in the TIC. Fragmentation of PI at *m/z* 488 resulted in two characteristic fragment ions at *m/z* 320 and *m/z* 169 (Fig. S10A). Comparing to the fragmentation of ZFB, fragment ion at *m/z* 320 indicates that no metabolic change in part B. Fragment ion at *m/z* 169 (28 *m/z* more) indicates the double oxidation at piperazine ring in part A (Fig. S10B).

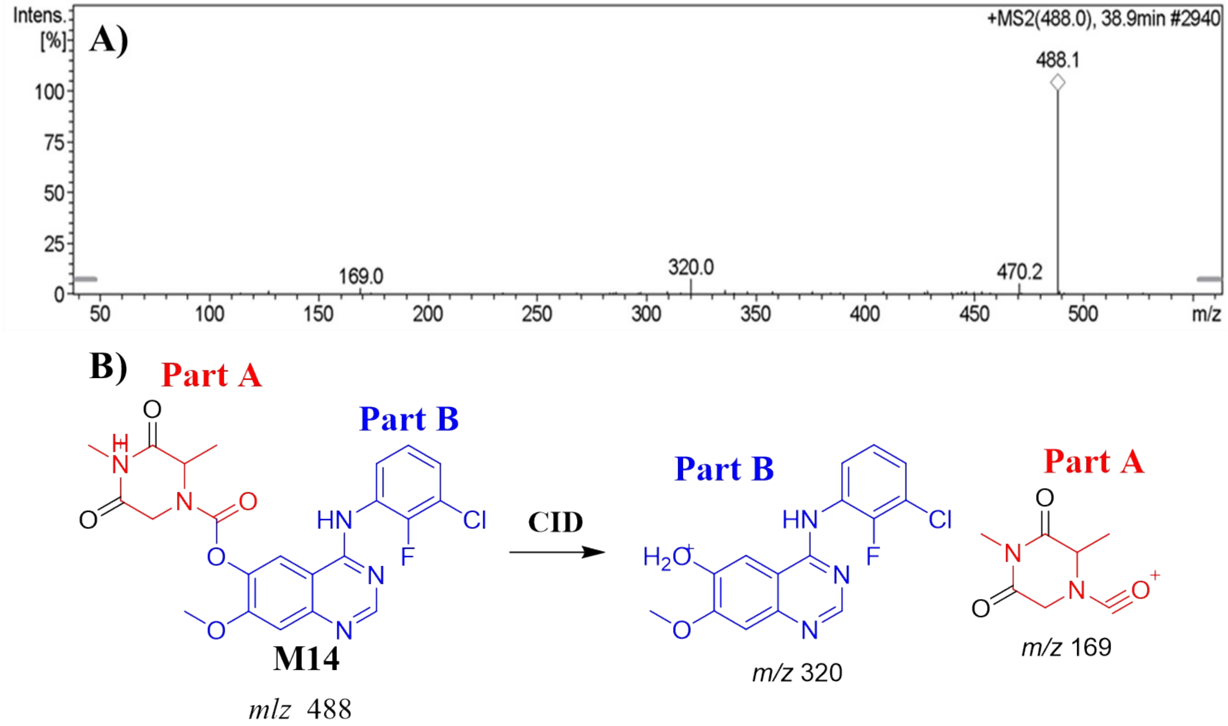

Fig.S10. MS^2 mass spectrum of M14 (A). Proposed M14 structure and its corresponding daughter ions (B).

4. *Cyano adducts of ZFB*

4.1. Identification of the AZDCN515 cyano adduct of ZFB

The AZDCN515 PIP appeared at 43.2 min. in the TIC. Fragmentation of PI at *m/z* 515 resulted in two characteristic fragment ions at *m/z* 488 and *m/z* 197 (Fig. S11A). Fragment ion at m/z 488 (27 *m/z* less) that indicates HCN loss and confirms the cyano adduct formation. Comparing to the fragmentation of ZFB, fragment ion at *m/z* 197 indicates that all metabolic changes occurred at part A including cyano nucleophile attack at bioactivated piperazine ring forming cyano adduct on parent drug in part A. Proposed metabolic changes at part A are α oxidation, N-methyl hydroxylation and cyano addition (Fig. S11B).

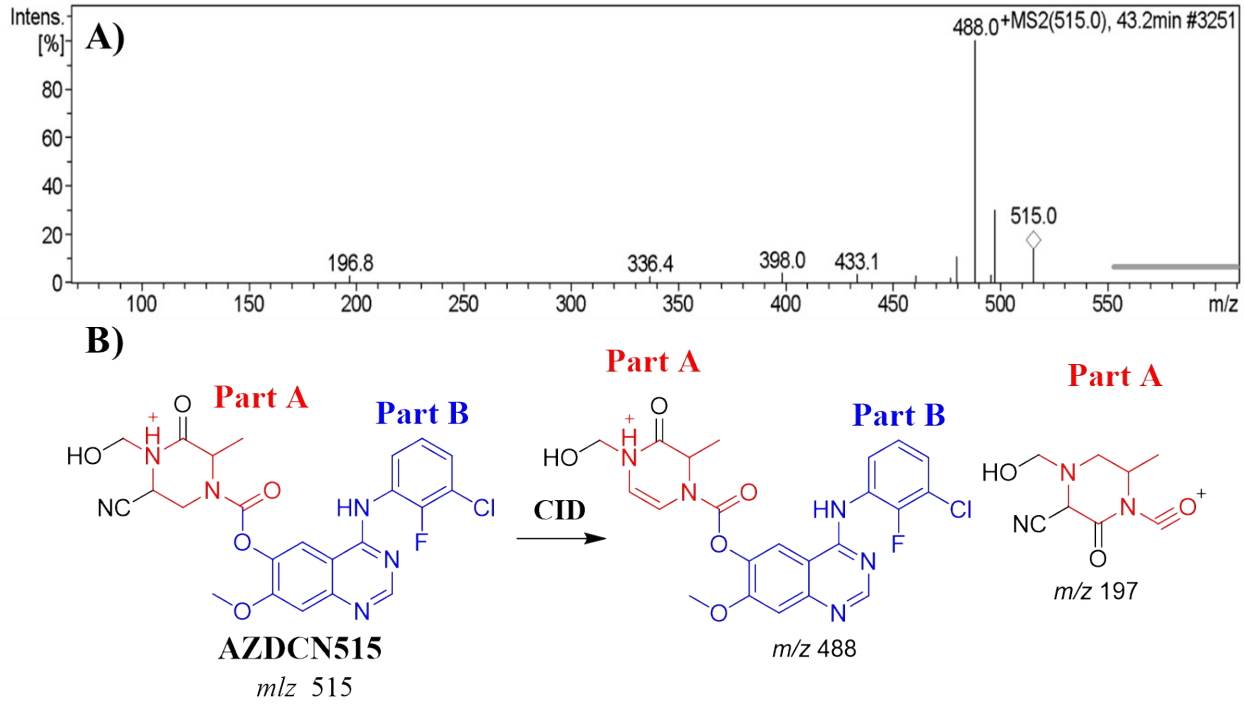

Fig. S11. MS[2] mass spectrum of AZDCN515 (A). Proposed AZDCN515 structure and its corresponding daughter ions (B).

4.2. Identification of the AZDCN501 cyano adduct of ZFB

The AZDCN501 PIP appeared at 45.5 min. in the TIC. Fragmentation of PI at *m/z* 501 resulted in two characteristic fragment ions at *m/z* 474 and *m/z* 327 (Fig. S12A). Fragment ion at m/z 474 (27 *m/z* less) that indicates HCN loss and confirms the cyano adduct formation. Comparing to the fragmentation of ZFB, fragment ion at *m/z* 327 shows water loss that confirmed hydroxylation metabolic reaction at N-methyl group. The proposed metabolic changes at part A are N-methyl hydroxylation and cyano addition that resulted from cyano nucleophile attack at bioactivated piperazine ring forming cyano adduct on parent drug in part A (Fig. S12B).

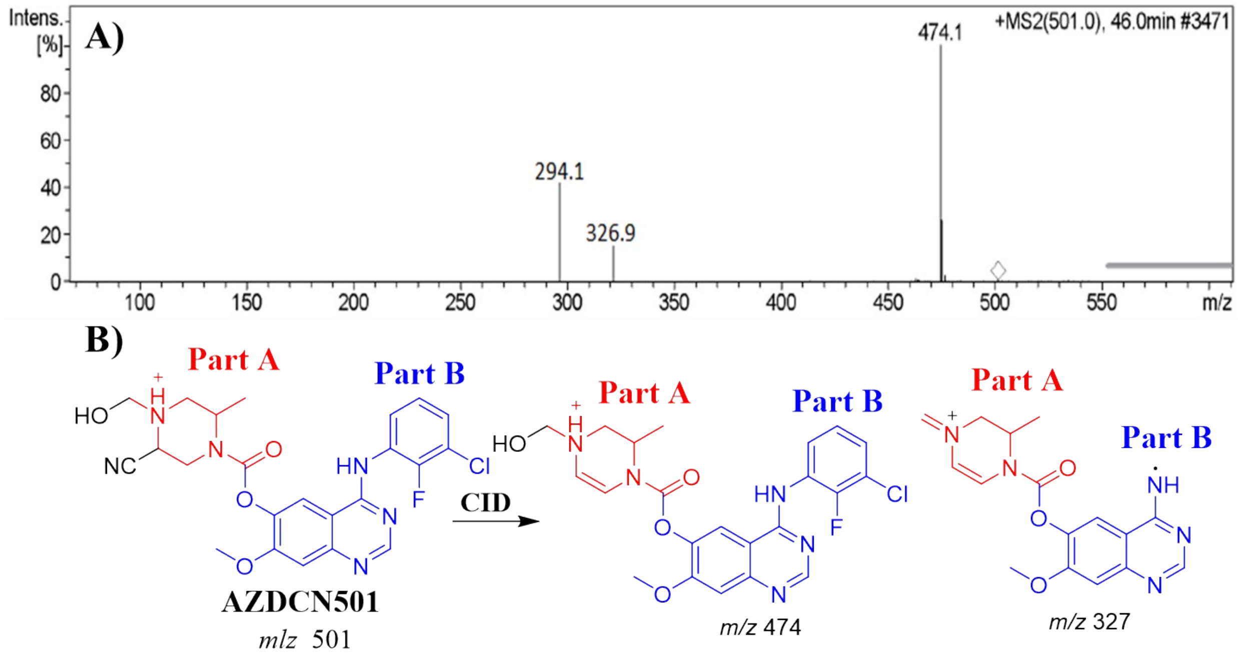

Fig. S12. MS^2 mass spectrum of AZDCN501 (A). Proposed AZDCN501 structure and its corresponding daughter ions (B).

5. GSH conjugates of ZFB.

5.1. Identification of the AZDGSH761 GSH conjugate of ZFB.

The AZDGSH761 PIP appeared at 43.1 min. in the TIC. Fragmentation of PI at *m/z* 761 resulted in two characteristic fragment ions at *m/z* 731 and *m/z* 632 (Fig. S13A). Fragment ion at m/z 632 (129 *m/z* less) that indicates anhydroglutamic acid moiety and confirms the GSH conjugate formation. Another LC-MS/MS screening for GSH adduct was performed by constant neutral loss scan monitoring of ions that lose 129 Da [28] (Fig. S13B). AZDGSH761 formation indicated the phenyl ring bioactivation in *in-vitro* metabolism of ZFB. The metabolic pathways that occurred in AZDGSH761 were proposed double hydroxylation at piperazine ring, dechlorination, defluorination, then hydroxylation, and then conjugation of GSH at bioactivated phenyl ring (iminoquinone derivative) (Fig. S13C).

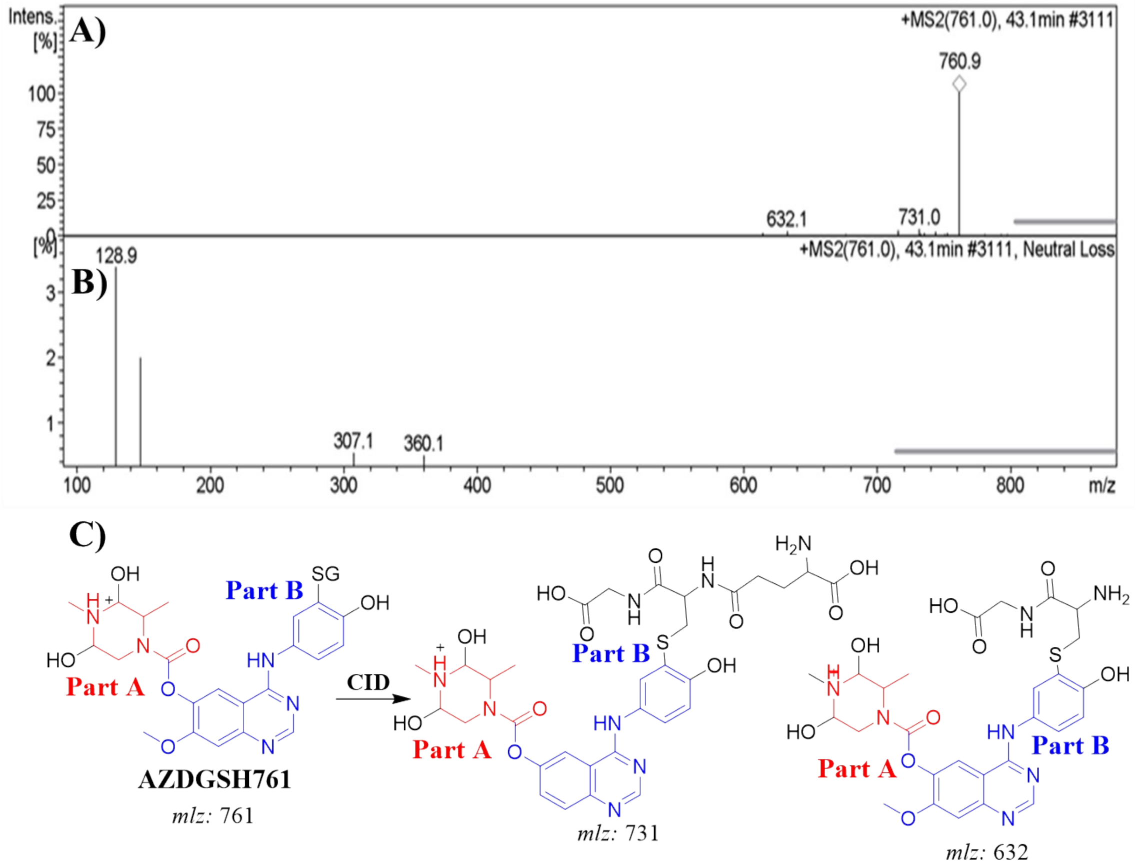

Fig. S13. MS² mass spectrum of AZDGSH761 (A). Constant neutral loss scan of AZDGSH761 (B). Proposed AZDGSH76 structure and its corresponding daughter ions (C).

5.2. Identification of the AZDGSH763 GSH conjugate of ZFB.

The AZDGSH763 PIP appeared at 36.4 min. in the TIC. Fragmentation of PI at *m/z* 763 resulted in two characteristic fragment ions at *m/z* 634 and *m/z* 458 (Fig. S14A). Fragment ion at m/z 634 (129 *m/z* less) that indicates anhydroglutamic acid moiety and confirms the GSH conjugate formation. Another LC-MS/MS screening for GSH adduct was performed by constant neutral loss scan monitoring of ions that lose 129 Da [28] (Fig. S14B). AZDGSH763 formation indicated the phenyl ring bioactivation in *in-vitro* metabolism of ZFB. The metabolic pathways that occurred in AZDGSH763 were proposed double hydroxylation at piperazine ring, reduction, dechlorination, defluorination, then hydroxylation, and then conjugation of GSH at bioactivated phenyl ring (iminoquinone derivative) (Fig. S14C).

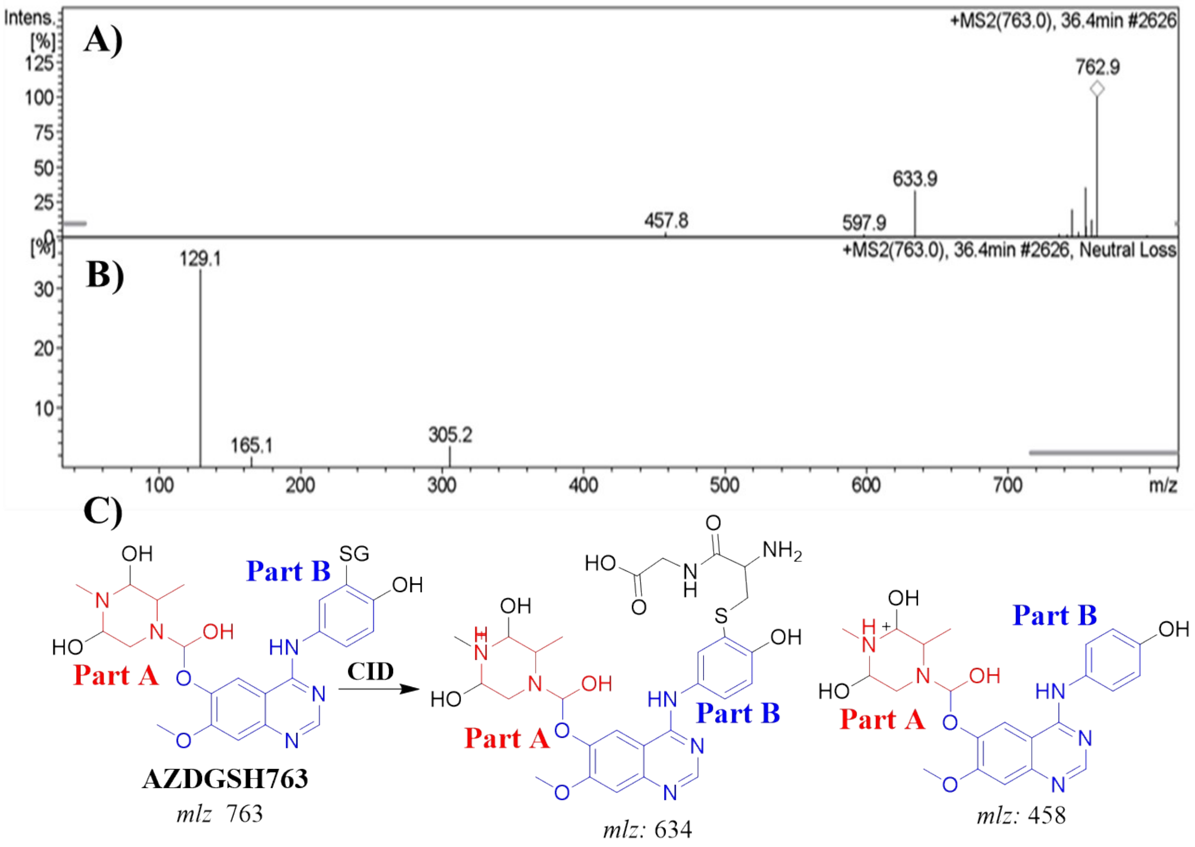

Fig. S14. MS^2 mass spectrum of AZDGSH763 (A). Constant neutral loss scan of AZDGSH763 (B). Proposed AZDGSH763 structure and its corresponding daughter ions (C).

5.3. Identification of the AZDGSH717 GSH conjugate of ZFB.

The AZDGSH717 PIP appeared at 31.2 min. in the TIC. Fragmentation of PI at *m/z* 717 resulted in two characteristic fragment ions at *m/z* 588 and *m/z* 307 (Fig. S15A). Fragment ion at m/z 588 (129 *m/z* less) that indicates anhydroglutamic acid moiety and confirms the GSH conjugate formation. Another LC-MS/MS screening for GSH adduct was performed by constant neutral loss scan monitoring of ions that lose 129 Da [28] (Fig. S15B). AZDGSH717 formation indicated the phenyl ring bioactivation in *in-vitro* metabolism of ZFB. The metabolic pathways that occurred in AZDGSH763 were proposed double hydroxylation at piperazine ring, *N*-demethylation, reduction, dechlorination, defluorination, then hydroxylation and conjugation of GSH at bioactivated phenyl ring (iminoquinone derivative) (Fig. S15C).

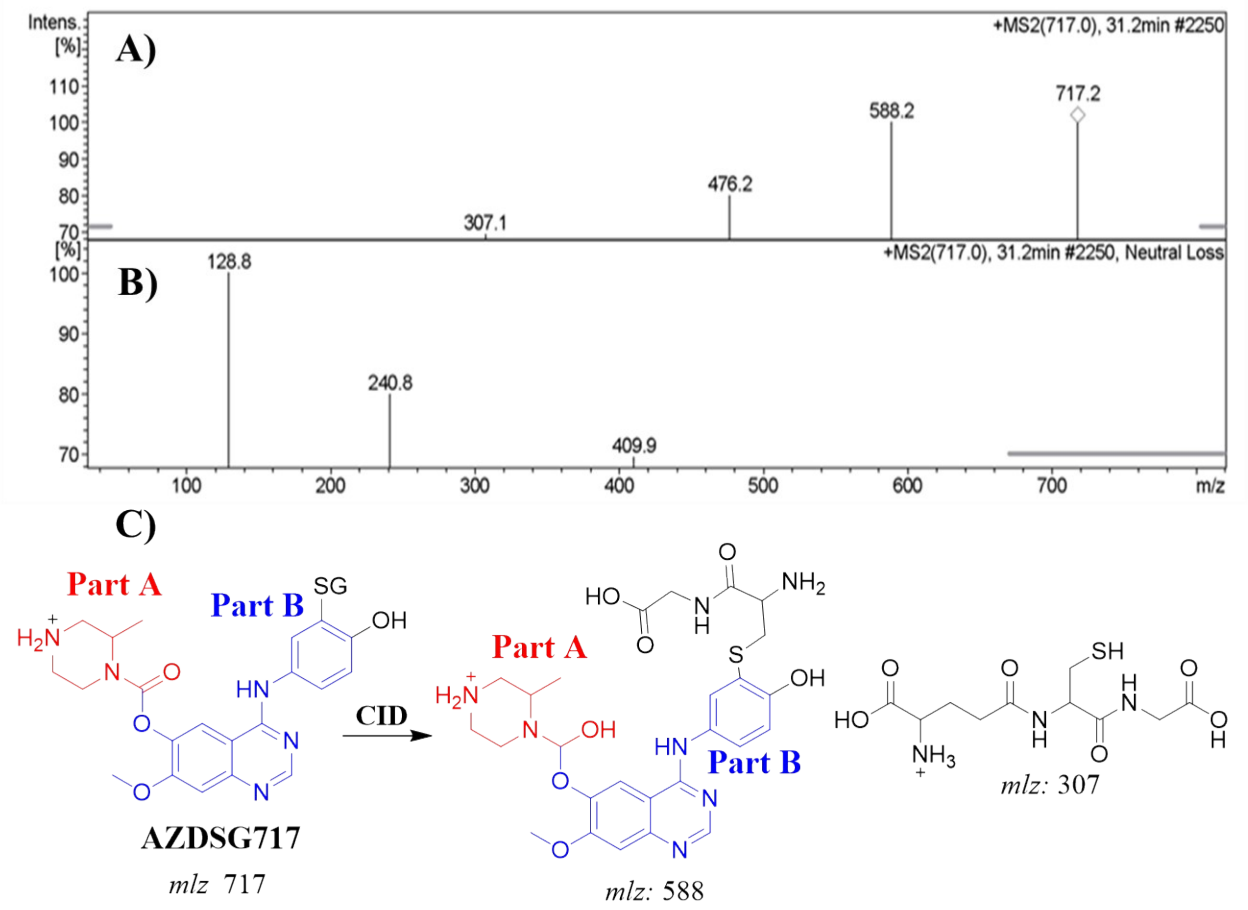

Fig. S15. MS^2 mass spectrum of AZDGSH717 (A). Constant neutral loss scan of AZDGSH717 (B). Proposed AZDGSH717 structure and its corresponding daughter ions (C).
